# Minnesota State Medical Society, Semi-Annual Meeting at Owatonna

**Published:** 1869-08

**Authors:** E. J. Davis

**Affiliations:** Secretary


### MINNESOTA STATE MEDICAL SOCIETY, SEMI-ANNUAL MEETING AT OWATONNA.

The State Medical Association convened at Owatonna, at 12 M., on the 16th inst., with Dr. J. H. Murphy temporarily in the Chair.

Minutes of last meeting read and approved. A committee of three on Credentials was appointed, when the convention adjourned until 2 P.M.

AFTERNOON SESSION.

Reassembled at the appointed time. Dr. Samuel Willey, President of the Society, arrived and took the Chair.

The Committee on Credentials reported the names of 17 physicians as duly qualified for membership, which report was accepted.

Dr. H. H. Kimball, the essayist appointed at the previous annual meeting, being absent, his valuable paper on "Rheumatism" was read by Dr. W. F. Hutchinson. An annimated discussion followed its reading, participated in by a number of gentlemen, upon points presented by the essay.

EVENING SESSION.

"Quackery," the subject for discussion was, on motion, changed to "Typhoid Fever." After the close of the debate, a resolution was introduced empowering the Chair to appoint a committee of three to report on Typhoid Fever, its origin, cause, and treatment, at the next annual meeting. The following gentlemen were appointed as such Committee:—Drs. Milligan, Richardson, and Mayo.

The President, Dr. Willey, offers for competition to members of the Society the two following prizes:—$50 for the best essay on Epidemic and Endemic Diseases of Minnesota, and $50 for the best essay on Cerebro-Spinal Meningitis. The respective merits of the essays to be determined by a committee appointed by the President.

A vote of thanks was unanimously adopted to the citizens of Owatonna, and especially Dr. Blood, for the generous hospitality extended by them to the members of the Society.

The Society then adjourned to the next general meeting at St. Paul, February 2d, 1870.

E. J. DAVIS, *Secretary.*

